# Anti-Tumor Effect of Adipose Tissue Derived-Mesenchymal Stem Cells Expressing Interferon-β and Treatment with Cisplatin in a Xenograft Mouse Model for Canine Melanoma

**DOI:** 10.1371/journal.pone.0074897

**Published:** 2013-09-09

**Authors:** Jin ok Ahn, Hee woo Lee, Kyoung won Seo, Sung keun Kang, Jeong chan Ra, Hwa young Youn

**Affiliations:** 1 Department of Internal Medicine, College of Veterinary Medicine, Seoul National University, Seoul, Republic of Korea; 2 Department of Internal Medicine, College of Veterinary Medicine, Chungnam National University, Daejeon, Republic of Korea; 3 Stem Cell Research Center, RNL Bio Co. Ltd, Seoul, Republic of Korea; Utrecht Universitym, Netherlands

## Abstract

Adipose tissue-derived mesenchymal stem cells (AT-MSCs) are attractive cell-therapy vehicles for the delivery of anti-tumor molecules into the tumor microenvironment. The innate tropism of AT-MSCs for tumors has important implications for effective cellular delivery of anti-tumor molecules, including cytokines, interferon, and pro-drugs. The present study was designed to determine the possibility that the combination of stem cell-based gene therapy with low-dose cisplatin would improve therapeutic efficacy against canine melanoma. The IFN-*β* transduced canine AT-MSCs (cAT-MSC-IFN-*β*) inhibited the growth of LMeC canine melanoma cells in direct and indirect *in vitro* co-culture systems. In animal experiments using BALB/c nude mouse xenografts, which developed by injecting LMeC cells, the combination treatment of cAT-MSC-IFN-*β* and low-dose cisplatin significantly reduced tumor volume compared with the other treatment groups. Fluorescent microscopic analysis with a TUNEL (terminal deoxynucleotidyl transferase-mediated nick-end labeling) assay of tumor section provided evidence for homing of cAT-MSC-IFN-*β* to the tumor site and revealed that the combination treatment of cAT-MSC-IFN-*β* with low-dose cisplatin induced high levels of cell apoptosis. These findings may prove useful in further explorations of the application of these combined approaches to the treatment of malignant melanoma and other tumors.

## Introduction

Malignant melanoma represents a significant and growing public health threat worldwide. The incidence of melanoma is rising [1] and deaths from malignant melanoma are increasing [2]. Surgical attempts at complete excision rarely are successful, and local recurrence is common [3], [4]. Once the disease becomes metastatic, standard chemotherapy has little effect [5]. As in humans, canine malignant melanoma is an aggressive and invasive neoplasm [3]. Complications from distant metastatic lesions such as those found in the lung, liver, and regional lymph nodes commonly occur [3], [6]. For these reasons, several alternative therapeutic strategies have been investigated [7]–[9]. In order to enhance the efficacy of melanoma therapy, a novel approach is required.

Mesenchymal stem cells (MSCs) are considered to be a promising platform for cell and gene therapy for a variety of diseases [10]. MSCs can routinely be isolated from several organs such as fetal liver, umbilical cord blood, bone marrow, and adipose tissue [11]–[13]. They have an extensive proliferative potential and the capacity to differentiate into various cell types. Compared to the other MSCs, adipose tissue-derived mesenchymal stem cells (AT-MSCs) are easier and simpler to isolate. AT-MSCs can be obtained in large quantities with a less invasive and less painful clinical procedure than that required for other types of MSCs. Importantly, the innate tropism of MSC for tumors makes these cells particularly effective for the cellular delivery of anti-cancer molecules including cytokines, interferons, or pro-drugs [14]–[16]. Moreover, the use of genetically-modified MSCs may represent an efficient alternative therapy capable of circumventing limitations associated with the systemic administration of some cytokines and drugs such as short half-life and toxicity [17]. Recent advances in the field of gene therapy have generated heightened expectations regarding the improvement of treatment for advanced malignancies, including melanoma [18], [19]. The cytokine interferon-beta (IFN-*β*) is known to have potent pro-apoptotic effects and is capable of inhibiting both tumor growth and angiogenesis [20]–[22]. Several reports indicate that mesenchymal stem cells engineered to secrete IFN-*β* trafficked to and reduced the tumor burden of melanoma, breast carcinoma, prostate cancer, and lung metastases [16], [23], [24]. Here, we investigated whether greater reduction of the tumor burden could be achieved by using targeted delivery of canine AT-MSCs (cAT-MSC) expressing IFN-*β* in combination with a low dose cisplatin (*cis*-diamminedichloroplatinum) protocol. Cisplatin is one of the most potent chemotherapeutic agents; unfortunately, it also often has significant gastrointestinal toxicities, nephrotoxicities, and hematological side effects [25]. However, the side effects of this drug are substantially reduced at a lower dose. It has been reported that the combination of IFN-*β* cytokine therapy with anti-cancer drugs synergistically suppressed the cell growth of hepatocellular carcinoma and melanoma [26]. Based upon this observation, we hypothesized that cAT-MSC-mediated targeted delivery of IFN-*β* might demonstrate a synergistic anti-tumor effect if combined with low dosage cisplatin.

In this study, we present evidence of a significant tumor suppression by cAT-MSC alone on canine melanoma (LMeC) *in vitro* and *in vivo* which was enhanced further when cAT-MSC expressed IFN-*β*. In addition, we investigated the effects of stem cell-mediated gene delivery of IFN-*β* in combination with systemic treatment with low doses of cisplatin in a canine malignant melanoma xenograft model; we found that this treatment combination resulted in a significant additive anti-tumor effect.

## Materials and Methods

### Cell isolation and culture

Canine adipose tissue-derived mesenchymal stem cells (cAT-MSCs) were isolated using modified methods previously described [27], [28]. Briefly, adipose tissue was collected from subcutaneous fat depots of Beagle dogs using standard surgical procedures. Each adipose tissue was digested overnight at 37°C with collagenase type IA (1 mg/mL; Sigma-Aldrich, St Louis, MO, USA) and then washed in phosphate-buffered saline (PBS). Following centrifugation, the pellet was filtered through a 100-µm nylon mesh and incubated overnight in Dulbecco's Modified Eagle's Medium (DMEM; Hyclone, Logan, UT, USA) supplemented with 10%, heat-inactivated fetal bovine serum (FBS; Hyclone) at 37°C in a humidified atmosphere of 5% CO_2_. After 24 h, non-adherent cells were removed by washing with PBS. The cell medium was then changed to K-NAC medium, which is a modified MCDB 153 medium (Keratinocyte-SFM; Invitrogen, Carlsbad, CA, USA) supplemented with 2 mM N-acetyl-L-cysteine (NAC; Sigma-Aldrich) and 0.2 mM L-ascorbic acid 2-phosphate (Asc 2P; Sigma-Aldrich). This medium contained 0.09 mM calcium, 5 ng/mL human recombinant epidermal growth factor (rEGF; Invitrogen), 50 µg/mL bovine pituitary extract (BPE; Invitrogen), 5 µg/mL insulin (Sigma-Aldrich) and 74 ng/mL hydrocortisone (Sigma-Aldrich). The medium was changed at 48-h intervals until the cells became confluent. When cells were >90% confluent, they were banked or serially subcultured under standard conditions. Before their use in the experiments, MSCs were identified based on the following cell surface markers: CD73^hi^, CD90^hi^, CD31^−^, and CD45^−^. The cAT-MSCs were maintained in DMEM supplemented with 10%, heat-inactivated FBS and 1X Pen/Strep (Invitrogen, CA, USA) at 37°C in a humidified atmosphere of 5% CO_2_. Canine AT-MSC preparation was performed under GMP (Good Manufacturing Practice) conditions (RNL BIO). LMeC, a canine melanoma cell line derived from metastatic mandibular lymph node of canine oral melanoma [29] was maintained in DMEM (Hyclone), supplemented with 10% FBS and 1X Pen/Strep at 37°C in a humidified atmosphere of 5% CO_2_.

### Construction of lentiviral vectors and transduction of cAT-MSC

The lentiviral vector carrying the canine interferon beta gene (cIFN-*β*) was generated as described previously [30]. Briefly, the target gene amplified by PCR from canine thymus cDNA was cloned into the pLenti6/V5-D-TOPO® vector using the pLenti/V5 Directional TOPO Cloning Kit (Invitrogen). The resulting lentiviral vector carrying IFN-*β* was sequenced to verify the correct reading frame and DNA sequence. Lentivirus particles were amplified in 293FT cells using the ViraPower™ Lentiviral Expression System (Invitrogen) according to the manufacturer's protocol.

For transduction, the viral supernatant was added to cAT-MSC at a multiplicity of infection of 5 with 6 µg/mLPolybrene (Sigma-Aldrich). After 16 h, the medium was replaced with fresh DMEM with 10% FBS. After an additional 24 h, the cells were cultivated in selection medium containing 5 µg/mL blasticidin (Invitrogen) for 5 days. The cells were prepared routinely and used for *in vitro* and *in vivo* studies as low-passage cultures (passages 4–6) [24].

Successful transduction of the cAT-MSC-IFN-*β* cells was confirmed by reverse transcription-PCR. The sense and antisense primers of each primer pair were designed to bind to different exons to exclude DNA contamination: canine IFN-*β* (sense 5′-GAGAGGATCCAATGACCAGTAGATGCATCCT-3′, antisense 5′-ATTTGATGTTGGCGGGAT-3′, 561 bp amplicon). Total RNA was extracted with easy-BLUE™ Total RNA Extraction kit (iNtRON Bio., Seoul, Korea). Complementary DNA templates from each sample were prepared from 1 µg of total RNA primed with oligodT primer using 400 units of Moloney murine leukemia virus reverse transcriptase (M-Mulv RT) (Invitrogen), followed by 30 PCR amplification cycles (94°C for 30 s, annealing at 57°C for 30 s, and extension at 72°C for 90 s). Glyceraldehyde-3-phosphate dehydrogenase (GAPDH) was used as the reaction standard: sense 5′-GGTCACCAGGGCTGCTTT-3′, antisense 5′-ATTTGATGTTGGCGGGAT-3′, 209 bp amplicon, 25 PCR amplification cycles). Each PCR product was analyzed by 1.5% agarose gel electrophoresis.

### IFN-*β* ELISA assay

The amount of IFN-*β* secreted by cAT-MSC-IFN-*β* into the media was quantified by a canine IFN-*β* enzyme-linked immunosorbent assay (ELISA) kit (BlueGene Biotech, Shanghai, China). cAT-MSC-IFN-*β* cells were plated at 1×10^5^ cells per well in 12-well plates. After 24, 48 and 72 h, the IFN-*β* level in the medium was determined according to manufacturer's protocols using recombinant IFN-*β* as a standard [31]. Assays were performed in triplicate.

### *In vitro* migration assay

The propensity of cAT-MSCs to migrate towards LMeC melanoma cells was evaluated using a modified 24-well-transwell migration assay. LMeC cells (10^5^ cells/mL) were incubated in serum-free DMEM for 24 h, conditioned medium was collected and placed in the lower wells of the transwell plates. Serum-free medium without any cells served as a negative control and medium supplemented with 10% FBS was used as a positive control. The cAT-MSC-Mock (empty vector-transduced cAT-MSC) or cAT-MSC-IFN-*β* (5×10^4^/250 µL) in serum-free medium were seeded onto transwell inserts (8 µm; BD Falcon) coated with gelatin (10 µL of 0.5 mg/mL). After incubation for 12 h at 37°C, the nonmigrating cells were removed from the upper surface of the transwell membrane using a cotton swab. The membranes were fixed and stained using 1% crystal violet (Sigma-Aldrich) in 4% Paraformaldehyde for 1 min and washed in distilled water. Nuclei of the migratory cells were counted in five high-power fields (×200). Results were expressed as the percentage of controls (cells migrating toward serum-free medium) (mean ±SD); all experiments were conducted in triplicate. The statistical significance in mean values among multiple sample groups was examined with two-way ANOVA and Bonferroni's *post-hoc* test using GraphPad Prism (version 4) software (Graphpad Software Inc., San Diego, CA, USA). Differences between two conditions at *p*<0.05 were considered statistically significant.

### Direct co-culture of LMeC melanoma cells with cAT-MSC *in vitro*

LMeC melanoma cells (3×10^3^ cells) were cultured either alone or mixed with 1.5×10^3^ cells of cAT-MSC-Mock or cAT-MSC-IFN-*β* (ratio of tumor cells: cAT-MSCs was 2∶1) on four-well chamber slides (Lab-Tek, Naperville, IL, USA) for 72 h. cAT-MSC-Mock and cAT-MSC-IFN-*β* cells were labeled with red fluorescent dye, CM-DiI (Molecular Probes, Eugene, OR, USA) before co-culture with LMeC cells. After incubation, LMeC cells and cAT-MSCs were stained with Hoechst 33342 (Lonza, Basel, Switzerland) to visualize the nuclei, and examined with a fluorescence microscopy Olympus BX41 microscope (Tokyo, Japan).

For flow cytometry (FACS) analysis, LMeC cells (1×10^5^ cells) were plated in 60-mm culture plates alone or mixed with cAT-MSC-Mock or cAT-MSC-IFN-*β*, respectively, at a ratio of 2 LMeC cells to 1 cAT-MSC-Mock or cAT-MSC-IFN-*β*. After 3 days, the cells were trypsinized, counted, and fixed with 70% ethanol. For analysis of DNA content, the cells were labeled with propidium iodide (Sigma-Aldrich) in the presence of RNase A (Sigma-Aldrich) (50 g/mL, 30 min, 37°C in the dark) [23]. Samples were run on a FACScan flow cytometer (Becton-Dickinson, FL, NJ, USA), and data were analyzed using FCS Express 4 (De Novo Software, Thornhill, Ontario, Canada). Results were expressed as the percentage of control cell growth: (the number of tumor cells co-cultured with cAT-MSC-Mock or with cAT-MSC-IFN-*β* on day 3 – the number of tumor cells co-cultured on day 0/(the number of tumor cells cultured alone on day 3 – number of tumor cells cultured alone on day 0)×100. The values were expressed as means ±SD. The statistical significance in mean values was examined with Bonferroni's multiple comparisons test after one-way ANOVA test.

### Indirect co-culture of LMeC melanoma cells with cAT-MSC and cell cycle analysis

A quantity of 5×10^4^ LMeC cells were plated on 6-well plate and 5×10^4^ or 2×10^5^ cells of cAT-MSC *β* (the ratio tumor cells∶cAT-MSCs was 1∶1 or 1∶4) were placed in transwell inserts (0.4 µm pore size; BD Falcon, Franklin Lakes, NJ). Inserts with cAT-MSC-Mock or cAT-MSC-IFN-*β* were transferred into wells with LMeC cells after 24 h of culture. After 3 days, the cells were trypsinized, counted, and fixed with 70% ethanol. For cell-cycle analysis, the cells were labeled with propidium iodide in the presence of RNase A (50 g/mL, 30 min, 37°C in the dark), and resuspension in PBS. Samples were run on a FACScan flow cytometer and the data were analyzed by using FCS Express 4. The results were the means (±SD) of three independent experiments. The statistical significance in mean values was examined with Bonferroni's multiple comparisons test after one-way ANOVA test.

### Evaluation of the effect of cAT-MSC-IFN-*β* on the growth of LMeC xenografts

**Figure 1:**
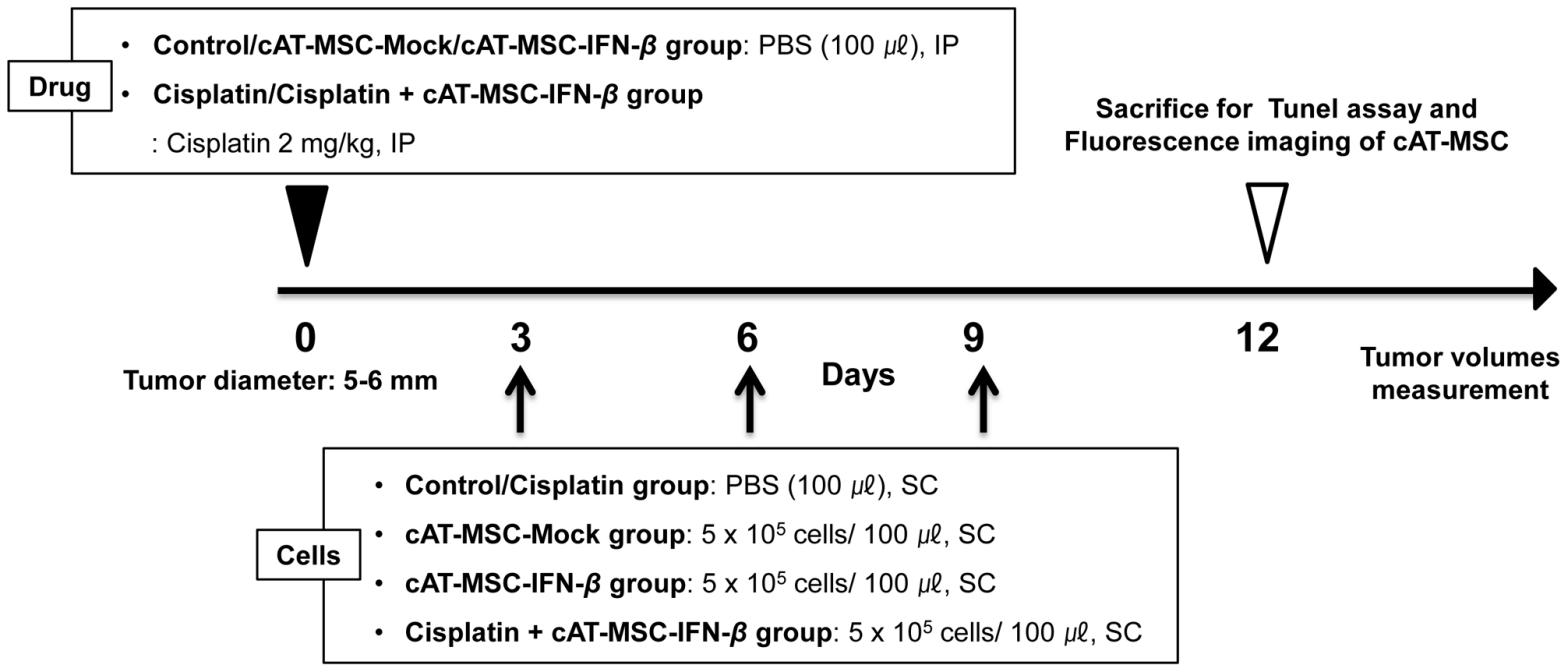
Time schedule for tumor therapy in the animal experiments. After tumor induction with LMeC melanoma cells (5×10^6^ cells), mice were randomly divided into five groups. The control group was treated with a circumtumoral injection of phosphate-buffered saline (PBS) (n = 4). The cisplatin and combination group were treated with intraperitoneal injection of low-dose cisplatin (2 mg/kg) (n = 4). The cAT-MSC-Mock, cAT-MSC-IFN-*β* and combination group were treated with a circumtumoral injection of each cAT-MSCs (5×10^5^) in PBS 3 times at 3 days interval. The size of each mass was measured every 3 days. Three days after the last injection, mice were sacrificed and tumor tissue was harvested for fluorescence microscopy analysis.

Five-week-old female BALB/c nude mice (20–30 g) were purchased from Central Lab. Animal, Inc. (Seoul, Republic of Korea). Mice were held for 1 week after arrival to allow them to acclimate. To induce canine melanoma tumor development in the animals, LMeC cells (5×10^6^) suspended in 200 µL PBS were injected subcutaneously (SC) into the flanks of mice. When tumors with a 5 to 6 mm diameter had developed, mice were randomly separated into five groups (n = 4/group), with each group receiving one of the following (Figure 1): Group 1 was given intraperitoneal, low dose cisplatin (2 mg/kg), group 2 was given circumtumoral cAT-MSC-Mock cells (5×10^5^/100 µL PBS), group 3 was given circumtumoral cAT-MSC-IFN-*β* cells (5×10^5^/100 µL PBS), group 4 was given a combination of intraperitoneal cisplatin (2 mg/kg) and circumtumoral cAT-MSC-IFN-*β* cells (5×10^5^/100 µL PBS), and group 5 was given circumtumoral PBS as a control. Three days after initiation of single drug treatment (PBS or cisplatin) cAT-MSC-Mock or cAT-MSC-IFN-*β* or PBS was administered 3 times at 3 days interval in respective treatment groups. The size of each tumor mass was measured every 3 days with a vernier caliper (Mitutoyo, Tokyo, Japan); tumor volume was calculated using the following formula: tumor volume (mm^3^) = (a^2^×b)/2, where a is the length of the short axis and b is the length of the long axis. The values were expressed as means ±SD. The statistical significance in mean values among multiple sample groups was examined with Newman-Keuls multiple comparisons test after one-way ANOVA test. Mice were euthanized when the tumors reached 3000 mm^3^ in volume (<10% of body weight), or as soon as tumors showed signs of necrosis, ulceration, or bleeding. Mice were killed by lethal exposure to CO_2_ followed by cervical dislocation.

### Tissue processing and imaging of transplanted cAT-MSCs

Homing of cAT-MSC to tumor tissue *in vivo* was determined by fluorescence microscopy analysis of cAT-MSC labeling with the red fluorescent dye, CM-DiI, before *in vivo* administration. Cultured cAT-MSCs were trypsinized and resuspended at a concentration of 1×10^6^ cells per 2 µg of CM-DiI dye in 1 mL of Dulbecco's PBS and labeled by incubation for 5 min at 37°C. Unincorporated dye was washed away with PBS then CM-DiI labeled cells were injected subcutanousely to mice exhibiting tumor formation. Three days after the last injection, mice were sacrificed and tumor tissue was harvested and fixed in 4% paraformaldehyde. Tumor tissue from each group of mice was embedded in Tissue Tek OTC compound (Sakura Finetek, CA, USA), snap-frozen in liquid nitrogen, and stored at −80°C. Frozen tissue was sectioned (7-µm-thick sections), mounted onto slides, and stained with Hoechst 33342. Images were captured with the use of a fluorescence microscope (IX 71, Olympus, Japan) equipped with a digital camera (DP71, Olympus) and processed using Image J software 1.45s version (National Institutes of Health, USA).

### TUNEL assay

Apoptotic cells in tumor tissue were identified using a TUNEL (terminal deoxynucleotidyl transferase-mediated nick-end labeling) assay (In Situ Cell Death Detection Kit, Fluorescein; Roche Diagnostics GmbH, Mannheim, Germany) according to the manufacturer's instructions. The 7-µm-thick frozen tissue sections were fixed with 4% paraformaldehyde for 20 min at room temperature followed by the addition of permeabilization solution (0.1% Triton X-100, 0.1% sodium citrate) for 2 min at 4°C. Labeling of DNA was done by treating the slides with 25 µL of TUNEL reaction mixture for 1 h at 37°C in a humidified chamber in the dark. The slides washed thrice in PBS and then the TUNEL-positive cells were analyzed under a fluorescence microscope.

### Ethics statement

All animals were handled in strict accordance with the recommendations in the Guide for the Care and Use of Laboratory Animals of the National Institutes of Health. The isolation procedure for cAT-MSC from beagle dogs was approved by the Seoul National University institutional Animal Care and Use Committee (Permit Number: SNU-130520-4). The mice study was approved by the Seoul National University Institutional Animal Care and Use Committee (Permit Number: SNU-110609-1).

## Results

### Confirmation of IFN-*β* expression from cAT-MSC-IFN-*β*

**Figure 2:**
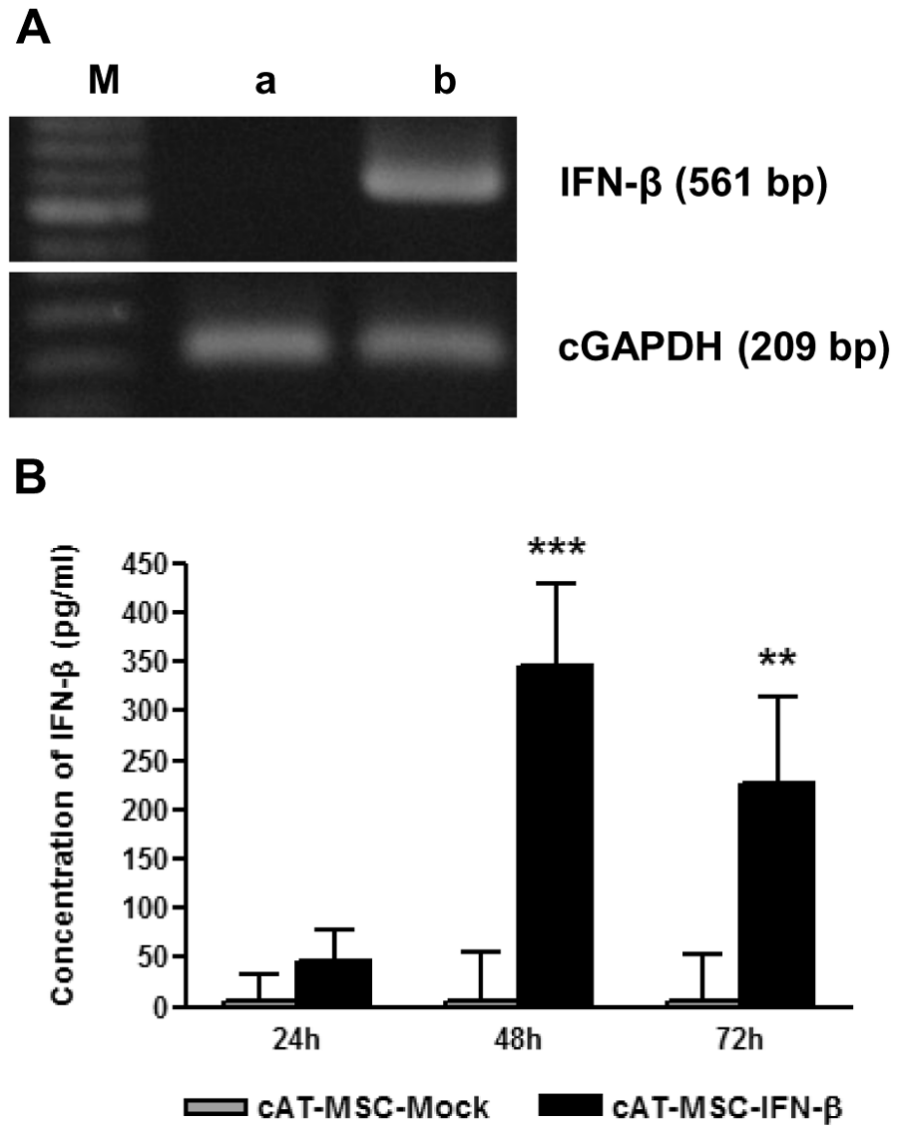
Confirmation of IFN-*β* expression in cAT-MSC-IFN-*β*. (A) RT-PCR analysis of canine IFN-*β* mRNA expression in cAT-MSC-IFN-*β* compared with cAT-MSC-Mock. The expressions of canine IFN-*β* and GAPDH (cGAPDH) were detected by reverse transcriptase (RT)-PCR, and GAPDH was employed as an internal control. M, 100-bp DNA size marker; a, cAT-MSC-Mock (empty vector transducted cAT-MSC); b, cAT-MSC-IFN-*β*. (B) Canine interferon-*β* concentration (pg/mL as mean +SD) by ELISA in conditioned media harvested after 24, 48 and 72 hours in cAT-MSC-IFN-*β* compared with cAT-MSC-Mock cells; statistically significant variation between the two cell types at ** *p*<0.01 or *** *p*<0.001.

cAT-MSCs were transduced with the canine IFN-*β*-expressing plasmid pLenti/V5 and its expression was confirmed by reverse transcription-PCR and ELISA. IFN-*β* mRNA expression was detected by RT-PCR, which showed positive expression only in cAT-MSC-IFN-*β* (Figure 2A). The resulting IFN-*β* protein expression was determined by ELISA and the cultured cAT-MSC-IFN-*β* supernatant contained a significantly greater amount of IFN-*β* protein compared with cAT-MSC control (Figure 2B). After 48 h of incubation, the canine IFN-*β* concentration produced by cAT-MSC-IFN-*β* was found to be 344.82 pg/mL or approximately 3.5×10^−3^ pg per cell. cAT-MSC-Mock cells had less than 5 pg/mL IFN-*β* protein expression.

### Migratory capability of cAT-MSC-IFN-*β in vitro*

**Figure 3:**
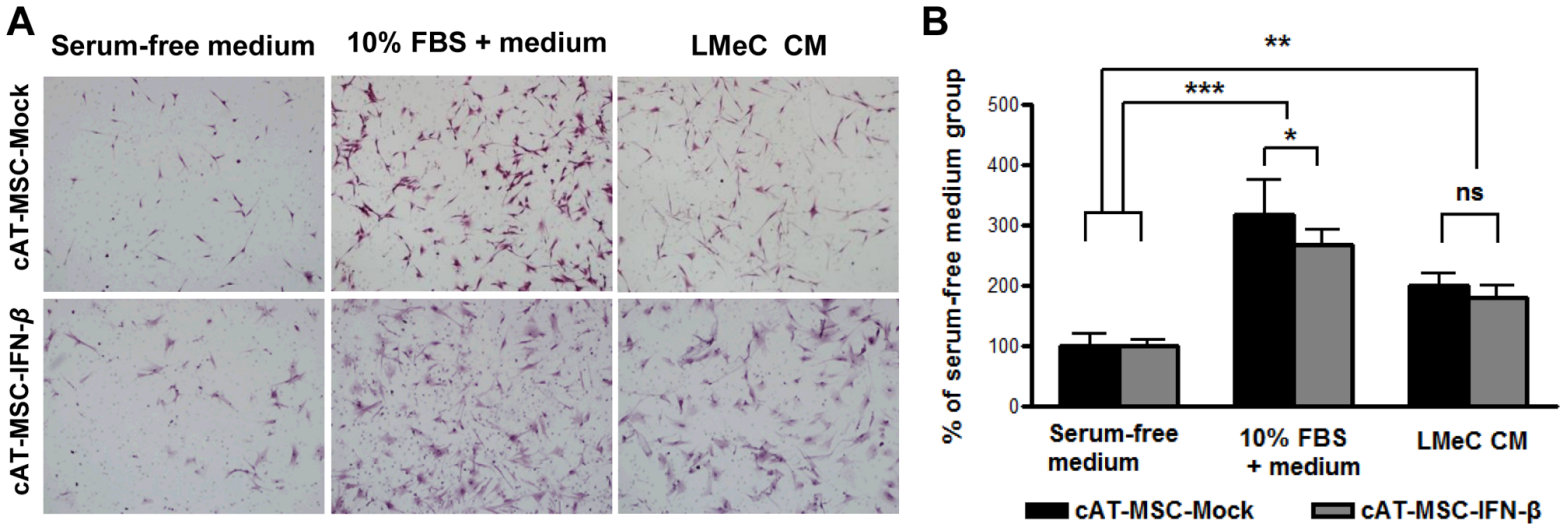
*In vitro* migration of cAT-MSC-Mock and cAT-MSC-IFN-*β* toward LMeC melanoma cells. The migratory capacity of cAT-MSC-Mock and cAT-MSC-IFN-*β* were assessed by a modified transwell migration assay. LMeC cells (10^5^ cells/mL) were incubated in serum-free DMEM for 24 h, conditioned medium was collected and placed in the lower wells of the transwell plates. Serum-free medium served as negative control and medium supplemented with 10% fetal bovine serum was used as a positive control. (A) Representative photographs (magnification, ×200). (B) The cAT-MSC-Mock and cAT-MSC-IFN-*β* showed significant migration toward LMeC conditioned medium (CM) compared with the serum-free medium control. There was no significant difference in migration ability toward LMeC CM between cAT-MSC-Mock and cAT-MSC-IFN-*β*. Values represent the mean +SD. Data are representative of three independent experiments with similar results. * *p*<0.05, ** *p*<0.01, *** *p*<0.001.

MSCs are intrinsically tropic for tumor cells which is central to their utility as a reliable delivery vehicle for cancer gene therapy [14]. The *in vitro* tumor-tropic properties of cAT-MSC-Mock and cAT-MSC-IFN-*β* to LMeC cells were evaluated using a modified transwell migration assay (Figure 3A and 3B). LMeC conditioned medium (CM) significantly stimulated migration of cAT-MSC-Mock and cAT-MSC-IFN-*β* as compared with negative control medium (*p*<0.01; Figure 3B). These two stem cell lines demonstrated significant migratory capabilities toward LMeC conditioned medium; however, there was no significant difference in migration ability toward LMeC CM between the cAT-MSC-Mock and cAT-MSC-IFN-*β* cell types.

### Inhibition of LMeC growth by cAT-MSC-Mock and cAT-MSC- IFN-*β in vitro*

**Figure 4:**
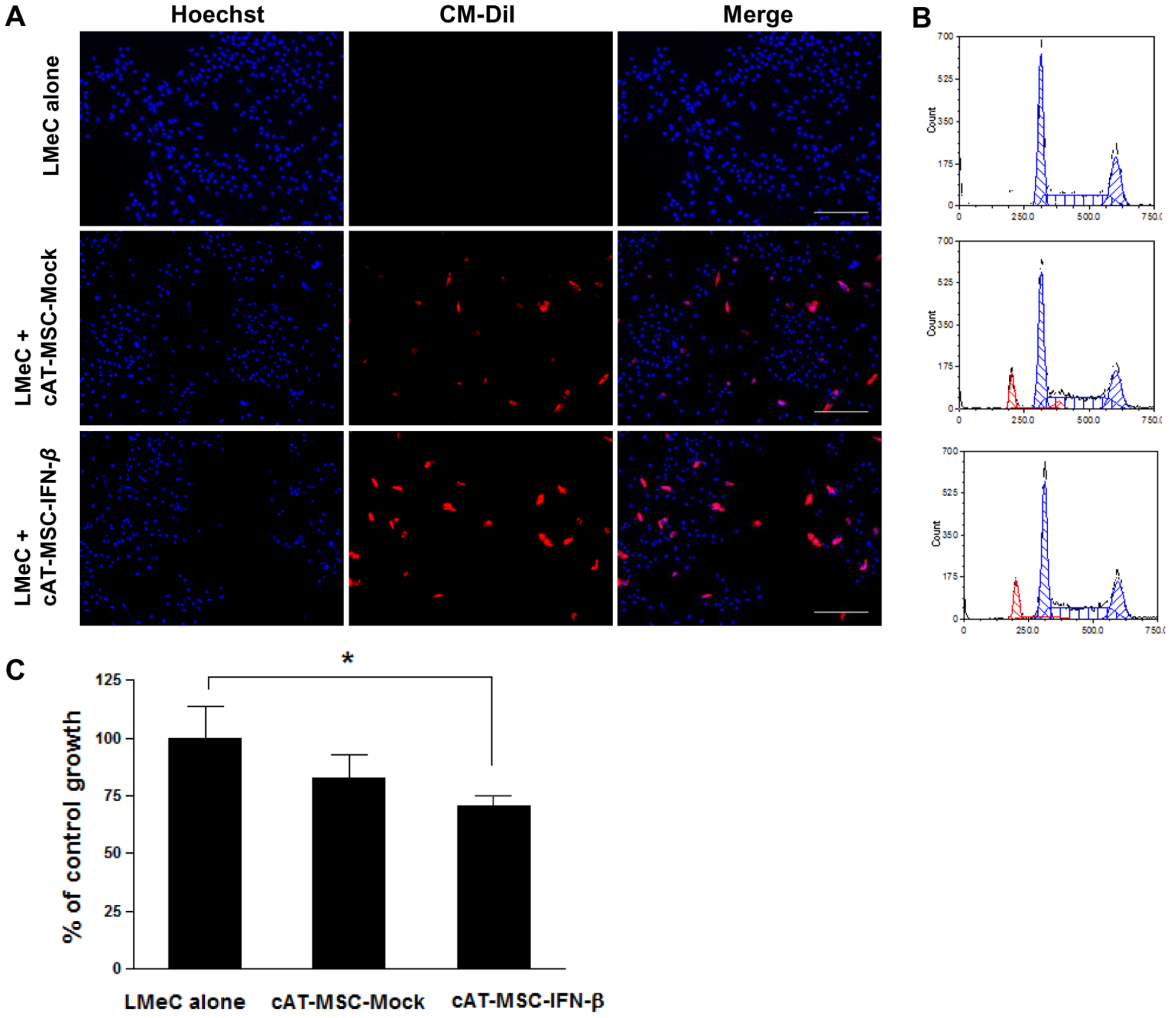
cAT-MSC-IFN-*β* directly inhibits the growth of LMeC melanoma cells *in vitro*. LMeC melanoma cells were either cultured alone or co-cultured directly with CM-DiI-labeled cAT-MSC-Mock and cAT-MSC-IFN-*β* (the ratio tumor cells∶cAT-MSCs was 2∶1) for 72 h. (A) LMeC cells and cAT-MSCs stained by Hoechst, were examined with fluorescence microscopy. Scale bar  = 100 µm. (B) Numbers of diploid MSCs (red) and aneuploid LMeC melanoma cells (blue) were determined by flow cytometry and (C) cell count. cAT-MSC-IFN-*β* directly inhibited the growth of LMeC cells as compared with LMeC cells alone. Data are expressed as the percentage of cell number and compared with that of the control. Values represent the mean +SD. Data are representative of three independent experiments with similar results. * *p*<0.05 (Bonferroni's method for multiple comparisons).

To investigate whether cAT-MSC-IFN-*β* cells have an inhibitory effect on LMeC cell growth and viability, LMeC cells were cultured at a 2∶1 ratio with CM-DiI-labeled cAT-MSC-Mock or cAT-MSC-IFN-*β* in a direct co-culture system (3×10^3^ LMeC to 1.5×10^3^ cAT-MSC-IFN-*β*). LMeC cells and cAT-MSCs stained by Hoechst, were observed by fluorescence microscopy (Figure 4A). As shown in Figure 4A, cAT-MSC-IFN-*β* directly inhibited the growth of LMeC cells as compared with LMeC cells alone. To evaluate the growth inhibitory effect of cAT-MSC-IFN-*β* on LMeC cells more accurately, cells were counted and the relative numbers of aneuploid tumor cells and diploid cAT-MSCs in the co-cultures were determined by flow cytometry (Figure 4B and 4C). After 72 h, the number of LMeC cells after co-culture with cAT-MSC-Mock or cAT-MSC-IFN-*β* had increased to 10.34±1.73×10^5^ and 8.40±0.55×10^5^ cells, respectively. In comparison, the number of LMeC cells in the control group (LMeC alone) had increased to 11.93±1.62×10^5^ cells. These data indicate that cAT-MSC-IFN-*β* can directly inhibit the growth of malignant tumor cells as compared to control (67.76% of control growth, *p*<0.05, Figure 4C), albeit in the absence of the host immune system. LMeC proliferation was slightly inhibited by co-culture with cAT-MSC-Mock, but the difference was not statistically significant (85.40% of control growth, Figure 4C).

**Figure 5:**
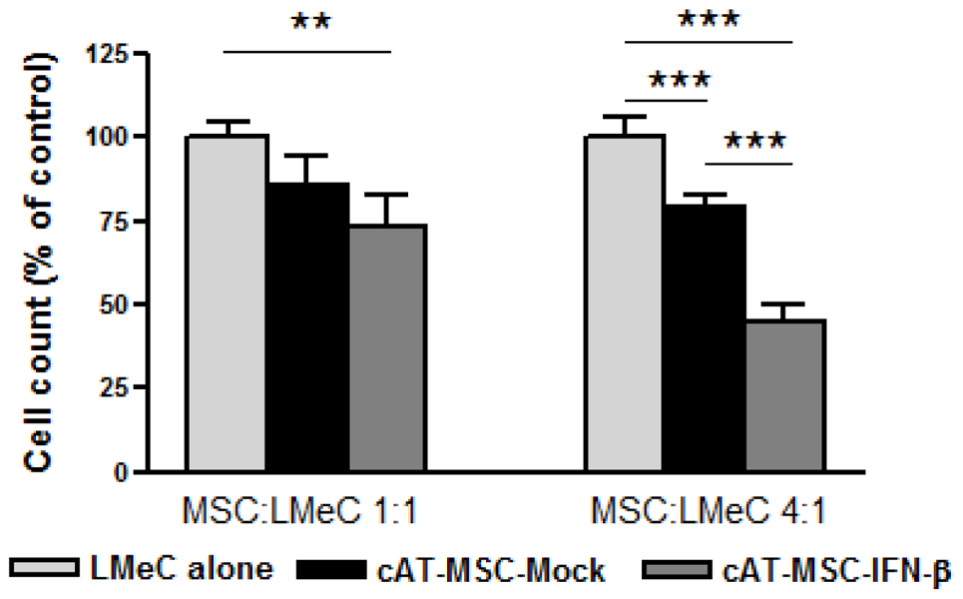
cAT-MSC-IFN-*β* inhibits the growth of LMeC melanoma cells in a transwell system. LMeC melanoma cells were either cultured alone or co-cultured with cAT-MSC-Mock or cAT-MSC-IFN-*β* for 72 h in a 1∶1 and 4∶1 (MSC/LMeC cell) ratio. LMeC cell growth was assessed with cAT-MSCs in a transwell format, which prevented LMeC-MSC cell contact. Data are expressed as the percentage of total cells compared with that of the control. Two cAT-MSCs exhibited a potent dose-dependent inhibitory effect on LMeC cell number. The P-value was obtained using one-way ANOVA with *post-hoc* Bonferroni's multiple comparison analysis. Each data point represents the mean +SD of three independent experiments. ** *p*<0.01, *** *p*<0.001.

Similarly, when cAT-MSC-IFN-*β* and LMeC cells were co-cultured but separated by a transwell membrane, which allows the exchange of soluble factors but prevents direct cell-to-cell contact, LMeC proliferation was again significantly reduced (Figure 5). The number of LMeC cells present after treatment with cAT-MSC-IFN-*β* was 9.85±1.31×10^5^ (1∶1 ratio, 73.23% of control growth, *p*<0.01) and 7.32±0.81×10^5^ (1∶4 ratio, 45.15% of control growth, *p*<0.001), respectively. In comparison, the number of LMeC cells in the control group was 13.45±0.66×10^5^ (1∶1) and 16.23±1.04×10^5^ (1∶4), respectively. The number of LMeC cells present after treatment with cAT-MSC-Mock was 11.5±1.25×10^5^ (1∶1 ratio, 85.5% of control growth, *p*>0.05) and 12.88±0.59×10^5^ (1∶4 ratio, 79.35% of control growth, *p*<0.001), respectively. The proliferation of LMeC cells was inhibited significantly when co-cultured with either cAT-MSC-IFN-*β* or with cAT-MSC-Mock (at a cAT-MSC/LMeC ratio of 4∶1). Therefore, expression of cAT-MSC-IFN-*β* exhibited potent dose-dependent inhibitory effects on LMeC cell proliferation.

### Effect of cAT-MSC- IFN-*β* on cell cycle distribution of LMeC

**Figure 6:**
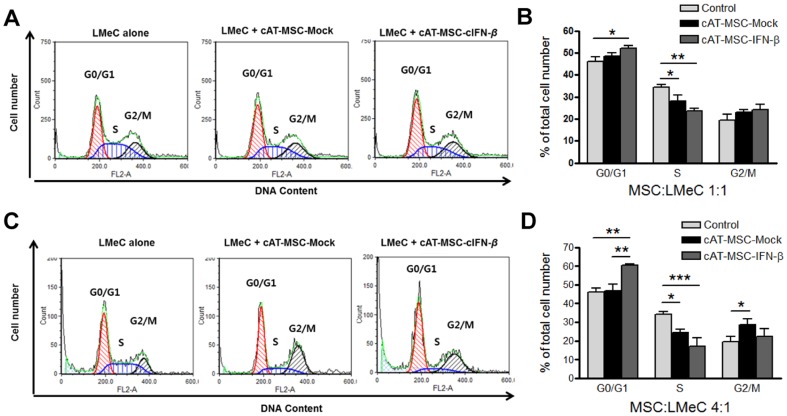
Cell cycle arrest of LMeC indirect co-cultured with cAT-MSC-IFN-*β*. The cell cycle phase distribution of LMeC was analyzed after harvest by flow cytometery. (A, B) cAT-MSC:LMeC 1∶1 and (C, D) 4∶1. The LMeC cells co-cultured with cAT-MSC-IFN-*β*, showed increase in the G0/G1 phase of the cell cycle compared to the controls (*p*<0.05 and *p*<0.01 at a cAT-MSC-IFN-*β*/LMeC ratio of 1∶1 and 4∶1 respectively). G1 arrest occurred concurrently with a reduction in the percentage of S phase cells (*p*<0.01 and *p*<0.001 at a cAT-MSC-IFN-*β*/LMeC ratio of 1∶1 and 4∶1 respectively). The P-value was obtained using one-way ANOVA with *post-hoc* Bonferroni's multiple comparison by comparing treated cAT-MSCs (cAT-MSC-Mock or cAT-MSC-IFN-*β*) with control (LMeC alone) for each phase of the cell cycle. Data are representative of three independent experiments with similar results. * *p*<0.05, ** *p*<0.01, *** *p*<0.001.

Flow cytometric cell cycle analysis showed that the proportion G0/G1 phase LMeC cells co-cultured with cAT-MSC-IFN-*β* at a ratio of 1 MSC to 1 LMeC cell, was higher than that of the controls (p<0.05, Figure 6A and 6B). LMeC cells co-cultured with cAT-MSC-IFN-*β* at a ratio of 4 MSCs to 1 LMeC cell, showed increases in the G0/G1 phase of the cell cycle compared to the controls (*p*<0.01, Figure 6C and 6D). G1 arrest occurred concurrently with a reduction in the percentage of S phase cells (*p*<0.01 and *p*<0.001 at a cAT-MSC-IFN-*β*/LMeC ratio of 1∶1 and 4∶1 respectively, Figure 6B and 6D). LMeC cells co-cultured with cAT-MSC-Mock also exhibited a decrease in S phase as compared to the controls (*p*<0.05 and *p*<0.05 at a cAT-MSC/LMeC ratio of 1∶1 and 4∶1 respectively, Figure 6B and 6D). LMeC co-cultured with cAT-MSC-Mock exhibited a slight increase in the percentage of cells in G2/M phase as compared to controls (*p*<0.05 at a cAT-MSC/LMeC ratio of 4∶1). These results show that cAT-MSC-Mock and cAT-MSC-IFN-*β* may prevent the normal progression of the tumor cell cycle.

### Effect of combination treatment of cAT-MSC-IFN-*β* and cisplatin on LMeC tumors *in vivo*

**Figure 7:**
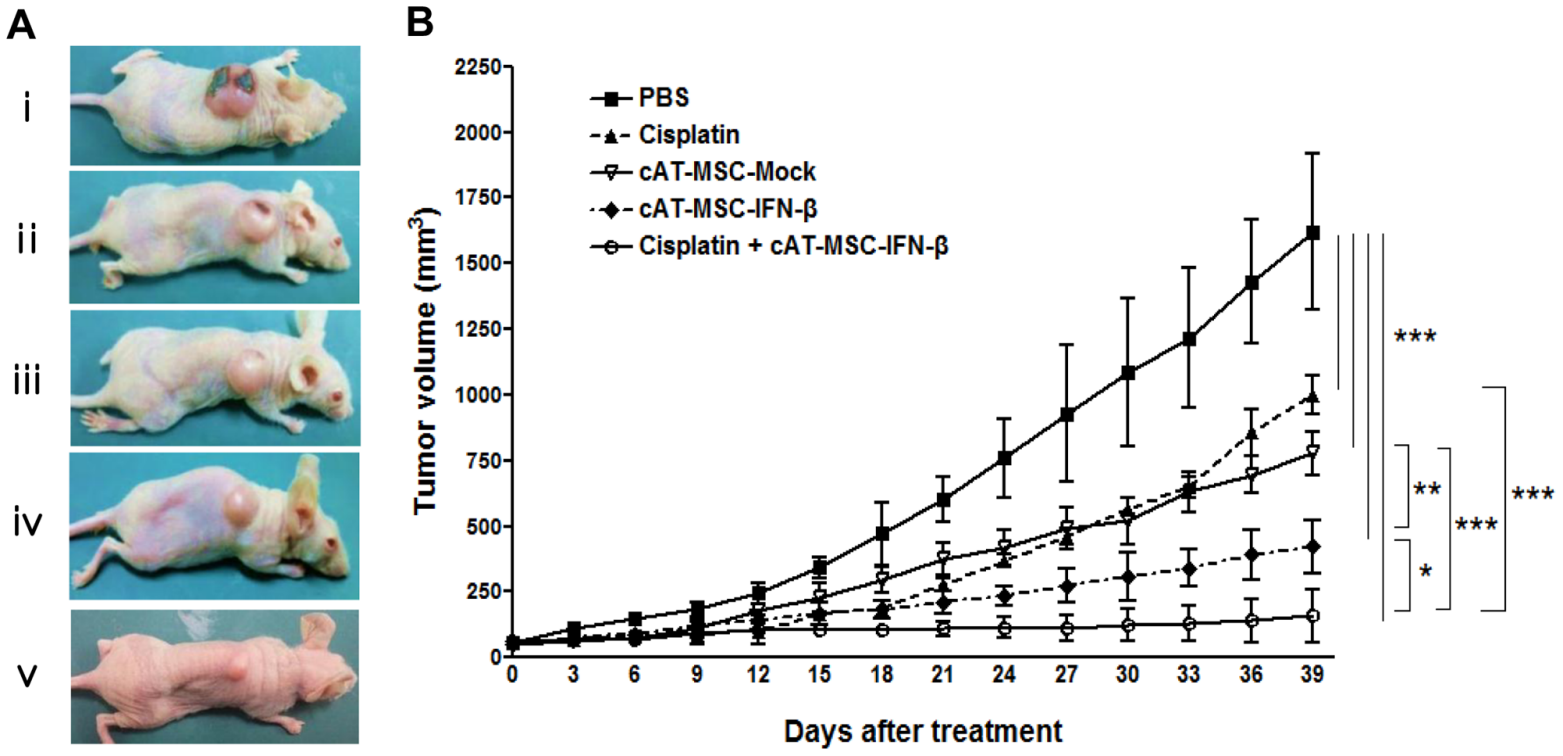
Growth inhibitory effects of cAT-MSC-IFN-*β* and low-dose cisplatin in a canine melanoma model. (A) Representative tumors on day 33 in athymic nude mice: i) PBS control; ii) Cisplatin (2 mg/kg); iii) cAT-MSC-Mock; iv) cAT-MSC-IFN-*β*; v) cAT-MSC-IFN-*β* combined with low-dose cisplatin (2 mg/kg). (B) Tumor volumes were measured in the LMeC melanoma model for the cisplatin-only, cAT-MSC-Mock-only, cAT-MSC-IFN-*β*-only and cAT-MSC-IFN-*β* combined with low-dose cisplatin (2 mg/kg). Animals (n = 4/group) were divided into five groups according to treatment: cisplatin (2 mg/kg), cAT-MSC-Mock (5×10^5^ cells), cAT-MSC-IFN-*β* (5×10^5^ cells) and cAT-MSC-IFN-*β* (5×10^5^ cells) combined with cisplatin. The size of each mass was measured every three days with vernier caliper. Tumor volumes were significantly reduced in all the groups that received either cisplatin, cAT-MSC-Mock, cAT-MSC-IFN-*β*,or both cisplatin and cAT-MSC-IFN-*β* cells, in comparison with mice that received PBS as a control. Mice that received combined treatment of cisplatin and cAT-MSC-IFN-*β* cells showed a greater reduction in tumor volume than the mice that received either cisplatin (*p*<0.001) or cAT-MSC-IFN-*β* cells alone (*p*<0.05). Data are presented as the mean ±SD, and determination of statistical significance was performed using a ANOVA analysis followed by Newman-Keuls multiple comparison test. * *p*<0.05, ** *p*<0.01, *** *p*<0.001.

To evaluate the antitumor effects of cAT-MSC-IFN-*β* combined with cisplatin on canine melanoma cells *in vivo*, LMeC cell xenografts were established in female BALB/C nude mice. Melanoma tumors were grown in nude mice by subcutaneous injections of LMeC cells into the flank. Control tumors grew rapidly, averaging 1620.4±298.4 mm^3^ in size by 39 days following initiation of treatment (Figure 7). In contrast, tumor volumes were significantly reduced in all groups that received either cisplatin (1000.8±72.6 mm^3^, *p*<0.001), cAT-MSC-Mock (777.4±84.0 mm^3^, *p*<0.001), cAT-MSC-IFN-*β* (421.1±102.1 mm^3^, *p*<0.001), or both cisplatin and cAT-MSC-IFN-*β* cells (157.7±98.7 mm^3^, *p*<0.001), in comparison with mice that received PBS as a control (Figure 7A and 7B). cAT-MSC-IFN-*β* produced a significantly greater tumor growth inhibition compared to cAT-MSC-Mock (*p*<0.01). Combining cAT-MSC-IFN-*β* with cisplatin produced a significantly greater tumor growth inhibition compared to cisplatin alone (*p*<0.001), cAT-MSC-Mock (*p*<0.001) or cAT-MSC-IFN-*β* alone (*p*<0.05). These data demonstrate that combination treatment can reduce the tumor volume more effectively than monotherapy.

### Transplanted cAT-MSCs migrate to melanoma tumor region

**Figure 8:**
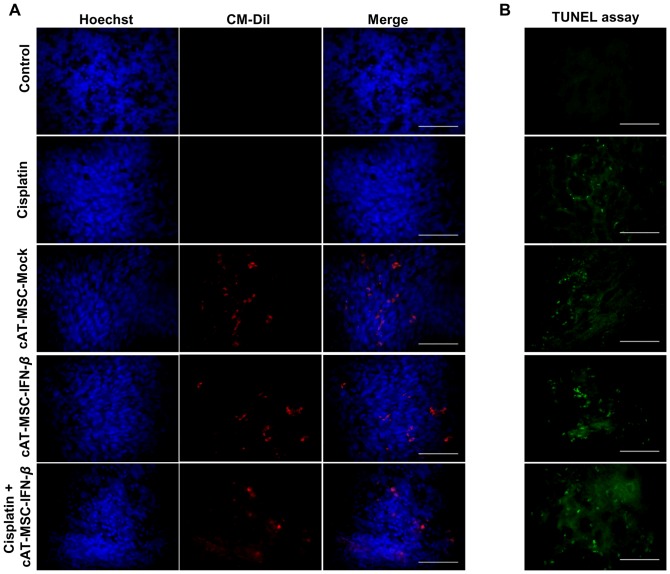
Fluorescent analysis of CM-DiI labeled cAT-MSC and apoptotic melanoma cells in the frozen tumor sections. (A) Fluorescent analysis of CM-DiI labeled cAT-MSC homing to tumors in the frozen tumor sections. Subcutaneously administered CM-DiI labeled cAT-MSC-Mock or cAT-MSC-IFN-*β* (red) integrate into tumor lesion. Sections were counterstained with Hochest 33342 nuclear stain (blue). (B) The combination treatment of cAT-MSC-IFN-*β* with cisplatin results in induction of apoptosis in LMeC melanoma model. Apoptotic cells were detected using the *in situ* death detection kit Fluorescein. Tumor tissues were harvested three days after the last injection of cAT-MSC. The combination treatment of cAT-MSC-IFN-*β* with cisplatin showed a greater apoptotic response than the other groups. Scale bar  = 25 µm.

To track the homing of transplanted cAT-MSC-IFN-*β* to tumor cells in the canine melanoma model, cAT-MSC-IFN-*β* cells were labeled with the cell tracker dye CM-DiI before *in vivo* administration. Three days after the last administration of cAT-MSCs in mice, the tissues were harvested and frozen tumor sections were made. Fluorescent microscopic analysis of tumor section provided evidence for homing of cAT-MSC-IFN-*β* (red fluorescence) to the tumor site as shown in Figure 8A. Therefore, cAT-MSC-IFN-*β* has tumor-tropic properties and localizes to melanoma tumor tissue where it exerts its therapeutic activity by producing IFN-*β*.

### Evaluation of tumor cell apoptosis by TUNEL assay

The induction of cell apoptosis in tumor tissues treated by each treatment group was evaluated by TUNEL assay (Figure 8B). Three days after the last injection of cAT-MSCs, representative tumors were harvested from each group and frozen tumor sections were made for subsequent apoptosis analyses. Combination treatment with cAT-MSC-IFN-*β* plus cisplatin resulted in a greater apoptotic response of cells than in the other treatment groups, indicating that enhancement of the apoptotic response may be contributing to the additive effect.

## Discussion

Conventional cytotoxic chemotherapy has been the mainstay of medical treatment for a variety of tumor types. However, clinical applications of chemotherapeutic agents are often limited by their dose-dependent toxicities and drug resistances [32]. Over the past decades, researchers have attempted to identify novel approaches to achieve more efficient melanoma therapies [33]. Although some therapies have reported promising preclinical results, clinical trials involving single-agent therapies have not indicated much benefit for patients' overall survival [34]. In this report, we found that additive effects could be achieved by a combination of stem cell-based gene therapy and chemotherapy in canine malignant melanoma. We show that cAT-MSCs selectively engraft in melanoma tissue, can be engineered to secrete a therapeutic protein, IFN-*β*, and can significantly reduce tumor burden in an animal model. To our knowledge, this is the first report to demonstrate the efficacy of combining a systemic chemotherapy with stem-cell-based, targeted delivery of a cytokine to a malignant canine melanoma in the athymic nude mouse.

We constructed a cAT-MSC expressing canine IFN-*β* using a lentiviral vector system, which offers the potential for long-term gene expression. AT-MSCs are considered to be a promising source of cellular vehicles for targeted cancer gene therapy [16], [24]. AT-MSCs have an intrinsic tumor tropism and can thus facilitate the local production of tumoricidal therapeutic agents within the tumor microenvironment. Previously, we confirmed that lentivirus-transduced cAT-MSC expressed the cell surface marker phenotype characteristic of AT-MSCs [11]. Indeed, flow cytometric analyses confirmed that cAT-MSC were positive for CD29, CD73, CD90, CD44 and CD105 yet lacked detectable CD31 and CD45 (unpublished data).

Next, we demonstrated the capability of cAT-MSC to actively migrate toward the tumor *in vitro* and *in vivo*. Both cAT-MSC-IFN-*β* as well as cAT-MSC-Mock demonstrated significant directional migratory capabilities toward LMeC cells, suggesting that the migration activity of cAT-MSCs was not influenced by lentiviral-vector-mediated genetic modification and IFN-*β* expression. The result of the migration assay also indicates that the melanoma cancer cells may contain chemoattractant factors which accelerate the migration of the cAT-MSC-Mock and cAT-MSC-IFN-*β* cells, thus enhancing the delivery of a therapeutic cytokine to tumors *in situ*. It has been reported that epidermal growth factor, platelet-derived growth factor, stromal cell-derived factor-1/CXCR4, SCF/c-Kit and vascular endothelial growth factor (VEGF)/VEGF receptor (VEGFR) 1 and VEGFR2 may play a role in the tumor-tropic effects [35]. Migratory properties of AT-MSCs should be further evaluated for tumor specificity and possible signaling mechanisms should be investigated in preparation for possible therapeutic applications.

Despite extensive investigation over the past ten years, the impact of MSCs on tumor progression is still greatly debated. Some studies have shown that MSCs promote tumor progression and metastasis [36], [37] yet other studies report that MSCs suppress tumor growth [38]–[40]. The reason for this discrepancy is unknown, but it may be attributable to differences in the experimental tumor models, the heterogeneity of MSCs preparations, the dose or timing of the MSCs injected, the animal host, or some as yet unknown factor [41]. The identification of the mechanisms involved in the interaction between stromal and cancer cells, especially the secreted factor responsible for the anti-proliferative effect of MSC is currently under investigation [38]. Inhibition of tumor growth may be mediated by high cytokine levels produced by MSCs. Other previous work has demonstrated an inhibitory effect on tumor growth mediated by MSC secretion of the Wnt-inhibitor, Dkk-1, which decreases cell cycle gene expression via the Wnt/*β*-catenin pathway [42], [43]. Tumor inhibition may thus be induced by down-regulation of positive cell cycle regulators, such as cyclin D1, D2 and CDK4, along with up-regulation of the negative regulator, cyclin dependent kinase inhibitor, p27, and its subsequent inhibition of Rb phosphorylation and G1 arrest [44]. As described in this communication, cAT-MSCs reduced LMeC cell viability and proliferation in an indirect co-culture system. Our observation that LMeC co-cultured with cAT-MSC-Mock exhibited a slight increase in the percentage of cells in G2/M phase as compared to controls is similar to the results reported by Ayuzawa *et al* [45]. We also observed that treatment with cAT-MSC-Mock reduced the tumor volume in tumor-bearing nude mice and induced an apoptotic response in tumor tissue. These results suggest that cAT-MSCs alone are capable of reducing growth of melanoma cells, perhaps by alteration of the cell cycle of cancer cells and stimulation of apoptosis.

All IFN molecules have antiviral and antiproliferative properties as well as some immunomodulatory activity. Considering both antiproliferative and anti-invasive effects of IFNs, IFN-*β* has the strongest anti-tumoral effect on human melanoma cells [46]. IFN-*β* may mediate anti-tumor effects either indirectly by modulating immunomodulatory and anti-angiogenic responses or directly by affecting proliferation or the cellular differentiation of tumor cells [47]. Despite these activities, clinical trials have failed to identify a clinical benefit for treatment with IFN-*β* [48]. These limited clinical results may result from the short half-life and the systemic toxicities of recombinant IFN-*β* protein at the doses needed to achieve an antitumor effect [48]. Because a local gene therapy strategy has the potential to surmount these limitations, we have tested the effect of IFN-*β* gene delivery by AT-MSC. We have carried out multiple *in vitro* experiments (cell ennumeration, flow cytometry and fluorescence imaging) in order to evaluate the anti-tumor effect of cAT-MSC-IFN-*β* on LMeC cells. Our study has revealed that IFN-*β*-transduced cAT-MSC secrete significant amounts of IFN-*β* (Figure 2B) and inhibit the growth of cancer cells in both direct and indirect co-culture systems (Figure 3 and 4).

Our data demonstrates that cAT-MSC-IFN-*β* have the ability to interfere with the proliferation of tumor cells by altering cell cycle progression. IFN-*β* can affect all phases of the mitotic cell cycle, most commonly via a block in G1 phase or, occasionally, by lengthening all phases of the cell cycle (G1, G2 and S) [49]. Although we did not test for the apoptotic effect of cAT-MSC-IFN-*β in vitro*, our *in vivo* data demonstrate that the treatment with cAT-MSC-IFN-*β* can induce an apoptotic response in tumor-bearing nude mice. These results are supported by several other studies in which IFN-*β* had an apoptotic and growth inhibitory effect on melanoma cells [16], [23], [30]. Induction of apoptosis by IFN-*β* involved FADD/caspase-8 signaling, activation of the caspase cascade, release of cytochrome c from mitochondria, disruption of mitochondrial potential, changes in plasma membrane integrity, and DNA fragmentation [22], [47].

The combination of traditional chemotherapy with IFN-*β* has been investigated for various cancers [26], [50], [51]. Eugene *et al.* reported that the combination of adenoviral-mediated IFN gene therapy and 5-fluorouracil resulted in tumor regression, apoptosis, and improved survival in an established liver metastases model [51]. The biochemical mechanism behind the synergistic effects of IFN-*β* with cisplatin or other chemotherapeutic agents are poorly understood. However, IFN-*β* is demonstrated to delay the cell cycle mainly in the S phase, which could affect the cellular uptake of chemotherapeutic agents [26]. Although this study does not show a synergistic anti-tumor effect of cAT-MSC-IFN-*β* with cisplatin on canine melanoma cells in vitro, we previously demonstrated the synergistic anti-tumor effects of cAT-MSC-IFN-*β* with cisplatin on mouse melanoma *in vitro* and *in vivo* [30]. Here, we found that combining stem cell-based IFN-*β* gene therapy with cisplatin showed greater reduction in canine melanoma burden than either treatment alone. Moreover, this combination strategy could make it possible to reduce the doses of chemotherapeutic agents and their accompanying systemic toxicities. As it is likely that chemotherapy will remain a mainstay of cancer therapy for many years to come, the combination of such chemotherapeutic agents with the stem cell-based gene therapy is likely to become an advantageous strategy.

Overall, we have demonstrated that cAT-MSC significantly attenuate the growth of LMeC canine melanoma cells in culture and in a mouse xenograft study, and can serve as cellular vehicles for the delivery and local production of anti-tumor agent. The anti-tumor effect is increased by when cAT-MSC express IFN-*β* and are combined with chemotherapeutic drugs. In conclusion, the present findings provide a strong rationale for the further exploration of the combination of AT-MSC with chemotherapy in the treatment of a malignant melanoma and other tumors.
